# Embedding patient and public involvement into a doctoral study: developing a point-of-care HIV testing intervention for dental settings

**DOI:** 10.3389/froh.2024.1359132

**Published:** 2024-05-15

**Authors:** J. Doughty, J. Preston, M. Paisi, A. Hudson, F. Burns, S. R. Porter, R. G. Watt

**Affiliations:** ^1^ Epidemiology and Public Health, University College London, London, United Kingdom; ^2^ School of Dentistry, University of Liverpool, Liverpool, United Kingdom; ^3^ Faculty of Health and Life Sciences, University of Liverpool, Liverpool, United Kingdom; ^4^ Peninsula Dental School and School of Nursing and Midwifery, University of Plymouth, Plymouth, United Kingdom; ^5^ Family Planning Association (FPA), London, United Kingdom; ^6^ UK Community Advisory Board (UKCAB), London, United Kingdom; ^7^ Infection and Population Health, University College London, London, United Kingdom; ^8^ Eastman Dental Institute, University College London, London, United Kingdom

**Keywords:** patient and public involvement, HIV, oral health, screening, public health

## Abstract

**Introduction:**

Patient and Public Involvement (PPI) can have a positive impact on research. PPI can make research more meaningful and appropriate as well as preventing research waste. For decades, patient advocates with HIV have played a key part in public health and research. This article presents the PPI activity undertaken during a doctoral study. The aim of this article is to demonstrate how PPI was embedded into a doctoral study that explored the feasibility of HIV testing in dental settings.

**Methods:**

Patients and the public were invited to be involved with the feasibility study through various organisations and charities. A comprehensive PPI activity strategy was devised, and appropriate funding was obtained. Patients and the public were predominantly consulted or collaboratively involved with several aspects of the study.

**Findings:**

Patients and the public positively contributed to the intervention development and the resources supporting its implementation. As a result, the study resources (i.e., questionnaire and information leaflets) were easier to read, and the intervention was more appropriate to the needs of patients. Furthermore, the training and focus groups conducted with dental patients and people with HIV benefitted from input of people with lived experience.

**Conclusions:**

PPI can be embedded within doctoral studies provided there is sufficient funding, flexibility, and supervisory support. However, PPI activity may be impacted by limited resource and *a priori* research protocol and funding agreements.

## Introduction

Globally there are 39 million people with HIV (PWH). Although the annual incidence of HIV has been decreasing in recent years, late diagnosis of HIV has remained a consistent global public health problem (1, 2). In the UK there are more than 100,000 PWH. Multiple approaches have been adopted to tackle late diagnosis of HIV, one of which has been the expansion of HIV testing into non-specialised health settings and community spaces (3). Dental practices have been identified as a promising setting for opportunistic testing for HIV (4). Dental professionals have successfully been trained to use finger prick and oral swab point-of-care tests and have implemented testing programmes in dental settings in the US and Canada (5–7). Patients report high levels of acceptability of HIV testing, particularly when these interventions are delivered in urban areas and in community dental clinics providing dental care for underserved populations (8, 9).

Patient and Public Involvement (PPI) can be a useful tool in designing interventions for health improvement to maximise their acceptability to the patient population. HIV activism ensured that PWH were involved and embedded in the design and conduct of much HIV health research. The civil society movements in HIV led to radical changes to the health research agenda and shaped the global AIDS response. For more than two decades, UNAIDS have recognised the value of HIV advocates’ involvement in research and have promoted the principal of Greater Involvement of People with AIDS (10–12).

Three key arguments in support of PPI are the normative, substantive and process perspectives. Normative (democratic) arguments consider PPI as important to upholding the values of justice, fairness, democracy and public accountability; a means to empower patients and the public (13). Alternatively, subjective (consequential) arguments position PPI in terms of its utility, consequences, or end outcomes for the benefit of research, for example, effectiveness, quality or relevance, validity or, representativeness. Process value systems are concerned with the conduct of PPI; this domain includes a focus on partnership, equality, respect, trust, openness, honesty, independence, and clarity (13). These approaches to defining the value of PPI are not at odds with one another, and can be complimentary e.g., with more equitable power sharing comes greater involvement and public accountability which can lead to improved quality of end outcomes.

In recent years PPI in healthcare research is increasingly well recognised and is now a mainstay of applications for research funding and ethical reviews. PPI is a key component of good research practice. Involvement of patients and the public are encouraged at all stages of the research process from concept through to dissemination and planning next steps (14). The benefits of PPI have been highlighted through improved retention and recruitment to clinical trials, benefits to study methodology and dissemination of innovations. Perhaps most importantly, PPI ensures that the interventions are tailored to meet the needs of participants and enhance the relevance and acceptability of interventions, thereby reducing research waste (15). However, alongside the burgeoning understanding of the value of PPI are concerns about tokenism and “box ticking”, thereby undermining the authenticity of the power sharing process fundamental to PPI (16).

Due to the wide variety of involvement strategies and different levels of involvement, it can be challenging to evidence the impact of PPI (17). Additionally, inconsistent reporting of PPI limits the usefulness, replicability and understanding of “how it works, in what context, for whom and why” (18).

There is an absence of literature describing how to operationalise PPI in doctoral studies (19). As a result, doctoral candidates may be discouraged from incorporating PPI in their research. Therefore, the aim of this article is to describe the approaches to PPI embedded into a doctoral study which explored the feasibility of implementing HIV testing in dental settings and to share the lessons learned throughout this process.

## Methods

Ethical approval was granted by the Essex, East of England, National Health Service Research Ethics Committee (IRAS 221512).

### The HIV dental study

The focus of the HIV Dental feasibility study was to design and implement an evidence-based point-of-care HIV testing intervention to be used in general dental practice and community dental settings. The mixed-methods feasibility study comprised of two phases. In the first phase, the intervention design was informed by a systematic review and focus groups undertaken with dental patients, PWH and dental professionals. In the second phase, the HIV testing intervention was introduced into dental settings and evaluated through a combination of clinical data, patient questionnaires, interviews with dental patients and professionals, and direct observation. The overall outcome was to ascertain whether HIV testing in dental settings was feasible and acceptable for implementation as a full-scale trial or roll out.

PPI activity was embedded within the intervention design process and was critical to the study literature development, focus group conduct and dental professional training aspects of the doctoral research study described in this article (22). PPI activity was planned into the grant application for the study and costed appropriately to enable the researchers to engage with patients and the public at multiple points. PPI activity planned for later aspects of the study was impacted by multiple contextual factors which are described in detail in the outcomes section of the manuscript.

We have applied the Guidance for Reporting Involvement of Patients and the Public—Short Form (GRIPP2-SF) checklist to the PPI activity undertaken as part of the doctoral study in order to provide rigour to the reporting process in this article (18). The GRIPP2 was developed to improve PPI reporting standards. It is the first international guidance for reporting patient and public involvement in health research. The checklist consists of two forms: short (SF) and long form (LF) versions. GRIPP2-SF includes five items and is primarily used for studies where PPI is a secondary focus. As per GRIPP 2 short form we have (1) reported the aim of PPI in the study, (2) provided a clear description of the PPI methods used in the study, (3) reported the outcomes of PPI, (4) commented on the extent to which PPI influenced the study positively and negatively, (5) reflected on what went well and what did not to enable others to learn from this experience.

### Patient and public involvement

For the purposes of this study we adopted the National Institute for Health and Care Research (NIHR) definition of PPI: “Research being carried out “with” or “by” members of the public rather than “to”, “about” or “for them”” (20). This definition is in line with other PPI-focused research papers published in the field of HIV prevention research.

There are five key approaches adopted by most PPI frameworks: power-focused, priority-setting, study-focused, report-focused, and partnership-focused. The PPI activity described in this article was study-focused (13). To that end, we attempted to build a culture of involvement at all stages of the intervention design process with an aim to improve the quality and appropriateness of the intervention. The PPI strategy was designed *a priori* with input from AH, who provided key insights both from his lived experience of HIV and past experience of leading national level HIV research studies e.g., the HIV stigma survey.

In this manuscript the level of PPI involvement is describe as either consultative, collaborative or user-led participation. Consultation takes place when researchers seek participant views to build knowledge and understanding of their lives and experience; they tend to be one off activities with no ongoing commitment. Collaboration affords more partnership between researchers and members of the public. for example, by enabling active engagement in resource design, undertaking research, policy development, and shared-decision making. User-led participation takes place where members of the public are empowered to initiate their own agenda and participate in self-directed engagement (21).

**Figure 1:**
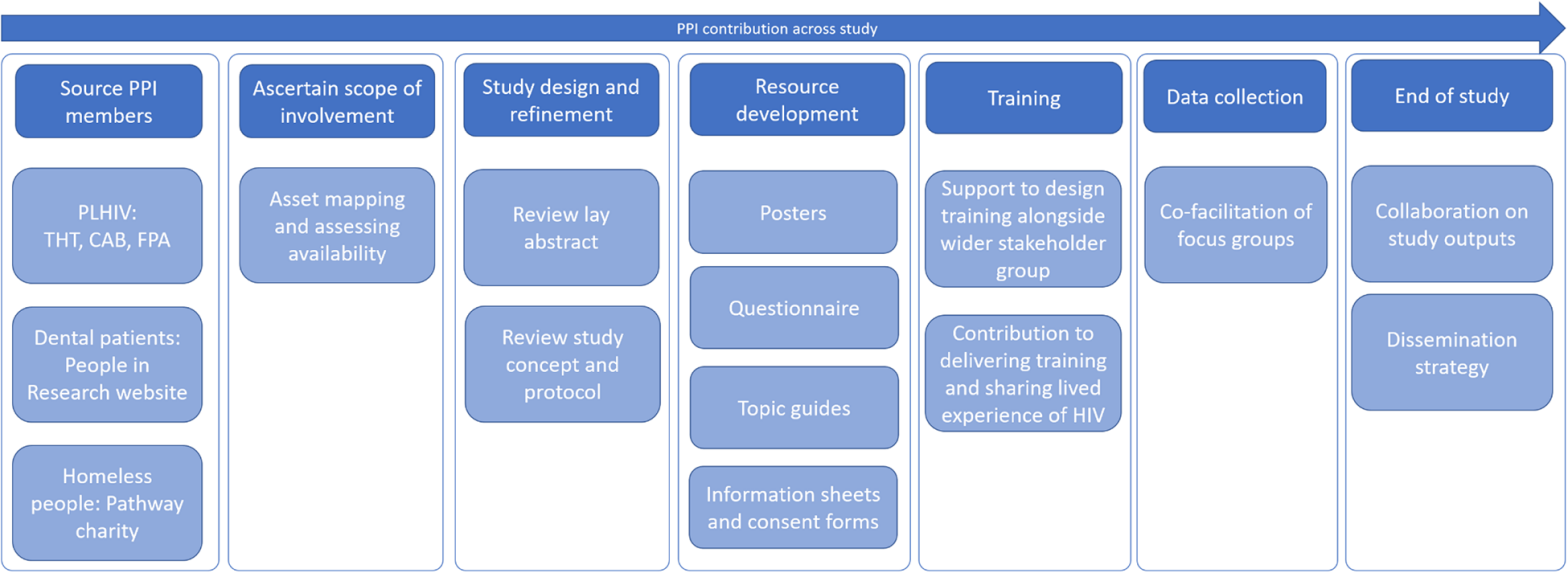
Stages of involvement of PPI during the doctoral study^1^.

An overview of the PPI activity embedded within the HIV Dental Study is presented in Figure 1. Information on PPI activities, who was involved and how they were involved is detailed in Table 1.

**Table 1:** Summary of patient and public involvement activity across the HIV dental study.

Aim of activity	Patient and public involvement details	Extent of engagement	Approach
Plain English Summary review and study rationale discussion (1 session)	Consulting with PPI group Patients in Research website, Terrence Higgins Trust, Family Planning Association, PPI contacts, Pathway Homeless Charity, Community Advisory board. Close working with AH throughout to understand the HIV landscape and the potential issues that could arise when implementing HIV testing in dental settings.	Consultation	Panel meetings face to face and online and email communication.
Introduction, asset mapping and availability questionnaire (1 session)	Formalising the study PPI advisory group from the above organisations.	Consultation	Face to face meetings. Email communication. Online questionnaire.
Developing study resources (3 sessions)	Three PPI members with lived experience of HIV, three people who had attended a dentist in the last twelve months and two people with lived experience of homelessness. Collaboration with wider study stakeholders including public health dentists and health researchers	Collaboration	Face to face meetings.Email communication.
Designing training (1 session)	PPI members (one PWH, one dental patient) and wider stakeholders for the study including general medical practitioner, health researcher and dental hygienist.	Collaboration	Hybrid method meetings. Email communication.
Delivering training	Co-delivering training. Identified key expert by experience with presentation skills through local sexual health services	Delivering a presentation, supporting role play activity	Face to face group session.
Data collection	Co-facilitating focus groups. Identified key expert by experience through Pathway Homeless Charity.	Collaboration	Face to face meetings.

### Researcher positionality

To provide further context to the reader, the study described in this article was undertaken by the lead author (JD). JD is a white woman and a dentist who was working in the clinical discipline of special care dentistry with a professional focus on providing dental care for inclusion health groups (e.g., people experiencing homelessness, sex-workers, drug users). At the time of conducting the study, JD was an NIHR-funded Doctoral Research Fellow (PhD student). JD was newly introduced to PPI in research through the grant application process and the subsequent PhD study that resulted from the approval of grant funding.

### Characteristics of PPI

The study PPI members had diverse characteristics which provided a range of perspectives for the study. PPI contributors included: (1) three people who regularly attended the dentist who did not have a known diagnosis of HIV, (2) three people who had lived experience of homelessness and had accessed homelessness-specific dental services, and (3) four people PWH. Within the group of PWH were heterosexuals, men who have sex with men (MSM), women, people of White and Black African ethnicity.

People with lived experience of homelessness were invited to contribute to PPI activity because the study was being conducted in two dedicated dental services for homeless people. PPI members were identified through the Pathway Homeless Health charity. The members of the PPI group who were living with HIV were contacted through the UK Community Advisory Board (UKCAB), Terence Higgins Trust (THT), and the Family Planning Association (FPA). Others were brought into the group through their contacts with the existing study PPI members with HIV. Specifically, a Black African woman who might not otherwise have considered PPI activity because of cultural stigma agreed to contribute after being invited to the study by a highly motivated PPI member.

Dental patients who had attended a dentist within the last 12 months were also invited as PPI members and were Identified through the People in Research website which advertises opportunities for public involvement in NHS, public health and social care research in the United Kingdom. Once the study funding had been awarded, PPI members were identified and invited to meet and greet sessions where the purpose and process of PPI was described.

Past experience of research varied among the PPI group. All but one of the PWH, two of the three people with lived experience of homelessness and one of the dental patients had previously been involved in research.

### Logistics and composition of PPI sessions

The location and timing of the PPI sessions were planned flexibly to accommodate the schedules of the individuals involved. The sessions were held in a private room within the grounds of University College London. The PPI members were reimbursed with £20 gift vouchers per hour of time; shop-specific gift vouchers could be requested. Transportation costs were covered through the study budget up to £15 Oyster travel card or PPI members could choose to phone in to the group meeting or speak over the phone individually (22). Each session lasted between one and two hours. In total, there were six PPI group sessions held to design the intervention and study resources, and two sessions where PPI attended to co-facilitate or deliver site intervention training. PPI members were often involved in mixed groups which comprised of PPI and wider stakeholders including a general medical practitioner, dentists, dental hygiene therapists, public health dentists, health researchers and sexual health professionals.

### Mechanisms supporting involvement and level of involvement

Level of involvement refers to the extent to which patients and the public were empowered to lead aspects of the study. The involvement matrix describes the extent of involvement in activities, processes, and decision-making. Involvement can take three different forms: consultation, collaboration or patient and public led/directed. Predominantly, throughout this study there was a process of consultation or collaboration. During the conceptual stages of the study, AH provided guidance and insight on study design, grant application content and HIV research funding bodies. The interaction with AH was fluid and flexible, consisting of face-to-face informal meetings, telephone conversations and email exchanges.

Once study funding was approved, PPI activity was conducted in a more formal and structured way. Communication with AH as the key PPI advisor remained regular and informal as well as his attendance at organised PPI meetings. At this stage asset mapping took place to identify the skillset already possessed by the PPI group, their willingness to be involved across a range of study activities and availability to attend meetings.

Activities were simplified to support engagement and facilitate involvement by all. For example, topic guides were cut up into individual questions and placed in piles of yes/no/maybe to indicate whether they were important to aims of the project or not. Posters that were to be used to recruit dental patients to test for HIV were designed by patients and the public who sketched out images to illustrate the types of pictures and text to use.

### Stages of involvement

In this doctoral study, PPI were involved from the conception of the study through to the intervention development and delivery of training (Figure 1).

At the grant application stage patients and the public reviewed the funding application and Plain English summary abstract; this was facilitated through UK CAB and THT/BASHH PPI panel. Once the grant application had been approved, an ongoing PPI group was set up to help design the study resources, intervention and training program.

Most PPI members expressed willingness to take part in piloting of the focus group topic guide, co-facilitating focus groups, contributing to dissemination and public engagement, and assisting with interpretation of transcripts from interviews or focus groups. Although PPI members were keen to be involved in all aspects of the study, the need for training was identified. Fewer than half of participants had experience of transcript analysis or designing study resources such as posters. JD was an inexperienced researcher in the early stages of the project and did not feel sufficiently knowledgeable to develop bespoke training in research methods for PPI. Unfortunately, this meant that involvement was limited in some of the latter research stages such as coding and thematic analysis.

PPI members were involved in discussion and refinement of the study protocol, adaptation, and production of the study resources, including information sheets, questionnaire, waiting room posters to recruit patients to test for HIV, and topic guides. Aspects of training which were supported by PPI activity included the development of: (1) a script advising dental professionals how to ask patients if they wished to have a HIV test, (2) presentation from the perspective of someone with lived experience of HIV and (3) PPI-supported role play activity designed to support dental professionals to offer HIV and manage the delivery of reactive test results.

PPI members were also involved with the wider research and experts’ team. For example, some PPI members chose to attend wider stakeholder group meetings to develop the training programme. The existing skills of PPI members were utilised by creating opportunities for co-facilitation of focus groups, delivering training sessions, and supporting role play sessions with dental professionals. Due to a number of contextual factors including maternity leave, end of study funding, PhD completion, and the Covid-19 pandemic led to a faltering of contact with PPI group members which is described in detail in the discussion.

### Measuring impact of PPI contributions

In the research literature, there is a lack of consensus about the best approach to defining and measuring PPI impact. In this study, the lead researcher (JD) created summaries of involvement sessions to record the ways in which PPI had impacted upon the study. After each PPI meeting, a session summary was sent to each attendee to highlight explicitly how their views and ideas had informed the study design and conduct. These session summaries were retained by the research team to evidence the contribution of the PPI members. No formal qualitative or quantitative processes were undertaken to measure the PPI impact on the study.

## Outcomes

Although there were multiple activities undertaken by PPI members, there were three overarching aims for the PPI involvement in the study:

1. Assess the appropriateness of the research topic and intervention design,
2. Review the paper-based study resources,
3. Support the practical delivery of aspects of the study (where feasible and appropriate to do so)

The impact of the PPI activity was documented using a “you said, we did” impact log, that was accompanied by researcher reflections on the process; similar approaches have been described in the PPI literature (23). Additionally, we did a PPI survey after each group session to understand to what extent PPI felt that their views had been listened to and acted upon.

### Assessing the appropriateness of the research topic and intervention design

PPI members expressed support for the intervention concept and understood the rationale behind the study. They explained that a key benefit of the intervention was that it provided another opportunity for people to test for HIV in a novel healthcare setting. Additionally, they felt that the dental setting could reduce the stigma associated with testing in sexual health settings, and that the focus should be on normalising testing.

Initially the research team had proposed the intervention was delivered by dentists. In contrast to this, the PPI group felt that it would be appropriate for any dental professional (including dental nurses and dental therapists or hygienists) to perform HIV testing. As a result, the intervention was adapted to invite the whole dental team to be involved in testing processes.

### Reviewing the paper-based study resources

PPI members reviewed several study resources including lay summary, patient-facing questionnaires, information sheets, consent forms and waiting rooms posters. PPI was fundamental to the grant application process. The Plain English summary was made easier to read using shorter paragraphs and sentences and language changes included describing test outcomes as “preliminary positive” rather than “reactive”. Other aspects of the patient-facing study resources that were changed because of PPI recommendations included:

- clarification around the cost implications for the HIV test,
- explaining that a universal (rather than targeted) approach to testing would be used,
- recognising national campaigns such as “can’t pass it on” and U = U^2^,
- changing the questionnaire language from “thinking” to “feeling” throughout,
- removing superfluous questions from topic guides and making the questions easier to understand.

To illustrate a crucial change to the study resources, a group of people with lived experience of homelessness chose to thoroughly redesign the study waiting room poster. The graphics changed from an empowered young person pointing a finger ready to test for HIV, to a person having an ordinary interaction with a dental professional sitting in a dental surgery chair. The poster wording changed accordingly. The ambition of the PPI members was to normalise the process of HIV testing at the dentist, as opposed to focusing on empowerment to test. The concept of normalising HIV testing had far-reaching implications for the study and ultimately contributed to the rationale behind adopting normalisation process theory as one of the key underpinning theoretical frameworks for the study.

### Involvement in practical aspects of the delivery of the study

Some PPI members were confident, engaged, and willing to lead or co-lead on aspects of the study. For example, one PPI member was willing to deliver a talk during the training session for dental teams. He devised, prepared, and delivered the talk independently, sharing his lived insight about HIV infection and the legal changes that support PWH e.g., The Equality Act 2010. Another PPI member agreed to co-facilitate a focus group with people with lived experience of homelessness. During the focus group, he felt confident to ask questions and to share his own experiences of homelessness. The importance of his presence during the focus group is exemplified by the following dialogue:

**Homeless male participant**: “*[…] I don't know what your experience of homelessness is.”*

**PPI facilitator**: “*I have experience of being a homeless person.”*

**Homeless male participant:** “*Oh, oh, now I'm going to ask other questions […] Were you on the street?”*

**PPI facilitator**: “*Yes, yes.”*

**Homeless male participant**: “*And for how long?”*

**PPI facilitator**: “*On and off. For like three years.”*

**Homeless male participant**: “*But do you actually think, I mean I don*’*t know who you mixed with or where you were…”*

**PPI facilitator**: “*I'll tell you exactly what I was thinking about, what I was thinking about was when I, because I was a junkie as well […] when I was sharing a spoon, with someone who had HIV, and I didn't have it. And I found that a couple of years later he died. You know what I mean, right?.”*

## Discussion

The PPI activity described in this study was wrapped around a doctoral research study. There is an absence of literature describing how to operationalise PPI in doctoral studies (19). Thus, this article provided a clear and transparent account of PPI within a doctoral study, highlighting the benefits and challenges.

PPI was important and influential to the PhD study design and conduct. It was a fundamental component of the successful grant application process and was recognised by the appraising research ethics board as a strength of the application. Input from PPI members led to changes in the wording of some resources and total redesign of others. Additionally, conversations with PPI members, changed the theory underpinning the intervention design for the study from an empowerment focus to normalisation of the intervention. As a result, involving patients and the public in the doctoral study enhanced the appropriateness of the study conduct and the resource design. Although many aspects of the study were limited to consultation, where it was feasible to do so collaboration and patient led aspects were supported.

Doctoral students have described barriers to PPI including additional planning, time, inadequate support from supervisors, funding for reimbursement and refreshments (19). In this study, PPI activity was scheduled into the grant application, supported by the supervisory team, and was costed for in the study funding budget, which enabled the researchers to engage with PPI at multiple points during the study. Hughes and Duffy (24) describe PPI activity on a conceptual spectrum from undefined involvement through to user-led research. Based on this typology, the PhD study described in this article progressed PPI activity beyond undefined or targeted consultation, to embedded consultation. Embedded consultation is characterised by regular consultation throughout the research cycle and the range of methods and people for consultation. However, the study did not progress PPI to co-production or user-led research.

PPI is reportedly more common in study types including mixed-methods, qualitative and intervention trial designs such as the study described in this article. However, PPI is less commonly applied in cohort studies or systematic reviews of analysis of secondary data (25). Though PPI activity was embedded into the interventional aspect of this study, it was not integrated into the systematic review that formed a crucial part of the intervention design process. The implications are that our narrative interpretation of the systematic review findings was limited to the researcher perspective and interpretation. In future studies, we would recommend PPI is embedded throughout systematic/literature reviews as part of good PPI practice for doctoral studies. In this way, PhD students can ensure that the follow-on components of their study are grounded in literature which has been collected and synthesised in a way that considers the lived experience of patients and the public and their priorities.

This PhD study illustrated pockets of PPI good practice by ensuring PPI was appropriately planned and costed and feedback was given to patients and the public about how their involvement had shaped the study. However, similar to other studies in primary care research, good practice in PPI was lacking in some areas. For example, PPI members were not involved in producing information for participants as the study progressed (e.g., writing blogs) or in interpreting the findings of the study (25).

Often the extent to which PPI members participated in the study conduct was limited to consultation rather than collaboration or enabling PPI to lead on aspects of the study. However, the fluidity of involvement across several aspects of the study enabled PPI members to contribute to the study in ways that were most meaningful and interesting to them. Further, PPI members were offered opportunities to meet with members of the wider study stakeholder team which enriched the discussion and created differing viewpoints.

An important barrier to involvement in some activities was the lack of availability of formal PPI training (e.g., co-facilitating, qualitative analysis). PhD researchers may be in the early stages of their research career, learning about and concurrently implementing research methods in their doctoral studies. As a result, PhD students may not feel confident to deliver bespoke PPI training on research topics which they themselves are in the infancy of competence building. Based on the experience of PPI throughout this doctoral study, our recommendations include providing PPI training to PhD student researchers at the beginning of their studies or in the lead up to application for doctoral funding programmes.

Though PPI activity underpinned the development of the protocol and research question, the power was not distributed uniformly in the researcher-PPI dynamic. As the study was undertaken as part of a Doctoral Research Fellowship, JD had overall responsibility to deliver the study and a PhD thesis within a set timeframe. These professional obligations to the University and funding body created barriers to equalising the power distribution in the researcher-PPI dynamic.

### Reflections and critical perspective

The following section critically reflects on practical and ethical issues encountered when PPI is undertaken within the independent study programme required of a PhD student

1. Power sharing between PhD researcher and PPI members

At times it was challenging to effectively distribute power equally between the PhD researcher and the PPI group amid competing priority of adhering to the study timeline. The PPI process added time to the preparatory stages of the study. Synthesising all the views from the stakeholders into a comprehensive list of study amendments and balancing these with the commitment to the study protocol took a considerable amount of time. Avoiding micro-managing and allowing PPI members ownership over their sessions was essential for the power sharing process. There were three PPI members who had pre-existing experience of co-facilitating, presenting, or writing scientific papers. In the absence of availability of PPI-specific training, the scope for involvement was limited for some PPI members. We provided transparency following all PPI activity by sharing a “you said, we did” impact log with all patients and the public and the wider team immediately after the meeting. In this way, we evidenced the important contribution of PPI to the study design and conduct. The study's PPI lead was also fundamental to the outputs from the research study and is recognised as a co-author on this manuscript. Three other key patients and the public who had actively contributed throughout the study were approached to co-author the manuscript but could not contribute at this time.

2. Equality, diversity, and inclusion in PPI

The NIHR Diversity and Inclusion group recommend strategies to ensure PPI activities are inclusive (26). In this study, we tried to ensure inclusivity by: reflecting on the power relationship between the researcher and people who may be from groups lacking power e.g., stigmatised or socially excluded populations. We valued the PPI group members contribution by providing a survey where they could highlight existing skills and areas of the study in which they wished to be involved. We used language carefully, avoiding jargon and were receptive to PPI feedback to simplify any scientific information that they found difficult to understand. We provided inclusive locations for meetings including university premises, local community centres or meeting rooms, and telephone/online alternatives. We collaborated with key community organisations including Pathway homelessness charity and multiple HIV charities to identify patients and the public to support the study. Through these approaches we were able to identify a diversity of voices of different genders, ethnicities and sexual orientations.

3. Finding ones’ feet in the early career researcher PPI journey

In the early stages of the study, supervisor mentorship and support was crucial to creating a bespoke PPI activity plan and to identifying the organisations to approach to source patients and the public. The supervisory team (FB, RGW and SP) provided regular and consistent guidance, whilst allowing the PhD candidate (JD) the space to develop PPI skills and to enact the PPI plans in a way that honoured her vision for the thesis. The supervisory team were readily available to consult on research methods for the duration of the study. Traditionally, doctoral students are expected to complete their work independently. This study challenges historical norms by highlighting the richness that lived experience can bring to the PhD research experience and the benefit it can have for deepening the early career researcher's understanding of the reality of living with health conditions such as HIV. Additionally, involving patients and the public in studies ensures that the PhD has relevance and is important to the target populations and is thus more impactful.

4. Limitations to the PhD researcher resource

As the study progressed and the focus moved toward enacting the practicalities of the study (e.g., implementation, data collection, and evaluation) communication with PPI members tailed off. There were a number of factors that impacted responsiveness and engagement. For example, the lead researcher conducting the study was doing so as part of a PhD; therefore, all PPI activity was coordinated by JD. During the implementation phase, two key things happened; firstly, JD broke from her studies during a period of maternity leave. Secondly, during maternity leave, the Covid-19 pandemic led to the early cessation of the study, major amendments to the study protocol and prevented face-to-face interactions. Upon returning from maternity leave, the focus of the study turned to practical analysis of study data and writing up of the PhD thesis within the available timeframe prescribed by the funding body. As a result, PPI activity ceased at this point in the study. Where PPI members were consulted on specific aspects of the study, the one-off nature of their involvement was explained from the outset. For the more actively engaged participants ongoing relationships and information-sharing were maintained through email communication or over the telephone.

5. PhD funding mechanisms

There was limited funding available for PPI activity prior to successful attainment of the study grant. Fortunately, existing PPI groups such as the Community Advisory Board and Terrence Higgins Trust, made possible the reviewing of the Plain English Summary and the proposed research questions and study design. Further, AH generously contributed his time due to his personal interest in bringing HIV testing in dental settings to fruition. Once funding had been confirmed, a new challenge emerged. Costings and plans for proposed PPI were submitted to the core funding body before the study commenced. However, as the study progressed, it became evident that more PPI input than was initially proposed would have been beneficial. This highlights the mismatch between the one-off funding application submission process and iterative approaches which might be required to fully involve patients and the public throughout the life of a project as new involvement needs emerge. Due to the temporal limitations of the study, all PPI members were made aware from the outset that the project timeline was restricted to the PhD funding envelope.

## Conclusion

This study demonstrates that even at an early stage in a research career, PPI can be integrated into doctoral studies and can encourage researchers to continue to consider PPI as they progress onward to research independence. PPI has the potential to benefit doctoral studies and offers an opportunity to familiarise the early career researcher with involvement processes. With sufficient funding, flexibility and support from the supervisory team, doctoral researchers can make their research more appropriate and acceptable through PPI activity. However, the responsibility for the research lies predominantly on the shoulders of the PhD student; therefore, if the researcher is compromised (e.g., during periods of maternity leave) PPI activity and continuity may be negatively affected.

## Data Availability

The original contributions presented in the study are included in the article/**Supplementary Material**, further inquiries can be directed to the corresponding author.

## Footnotes

^1^Terrence Higgins Trust (THT), Community Advisory Board (CAB), Family Planning Association (FPA).

^2^Undetectable=untransmissible (U=U). U=U means that people with HIV who achieve and maintain undetectable viral load cannot sexually transmit the virus to others.
