# Supplementary material for: Embedding patient and public involvement into a doctoral study: developing a point-of-care HIV testing intervention for dental settings
Source: Front Oral Health. 2024 May 15;5:1359132. doi: 10.3389/froh.2024.1359132 (PMC11133536; doi:10.3389/froh.2024.1359132)

Supplemental material

Supplemental figure: Poster before (top) and after (bottom) patient and public involvement


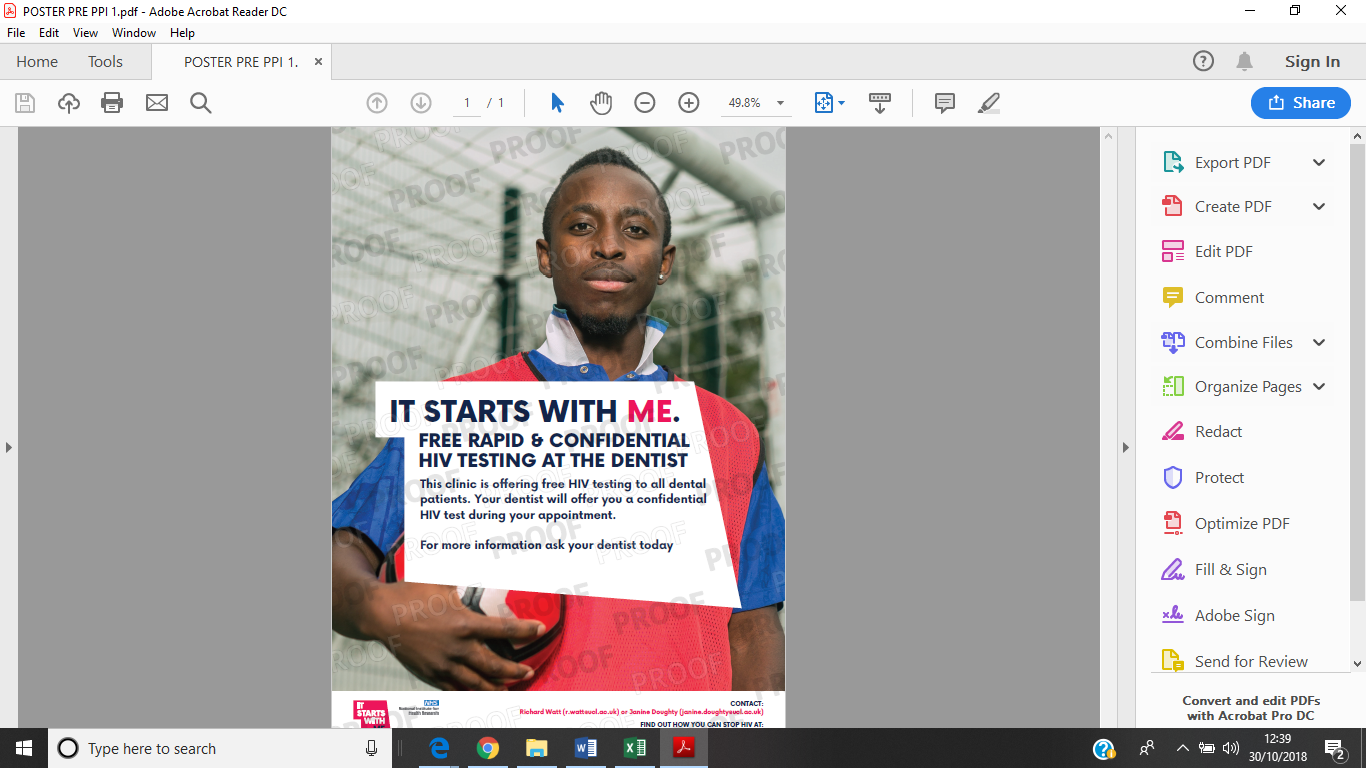


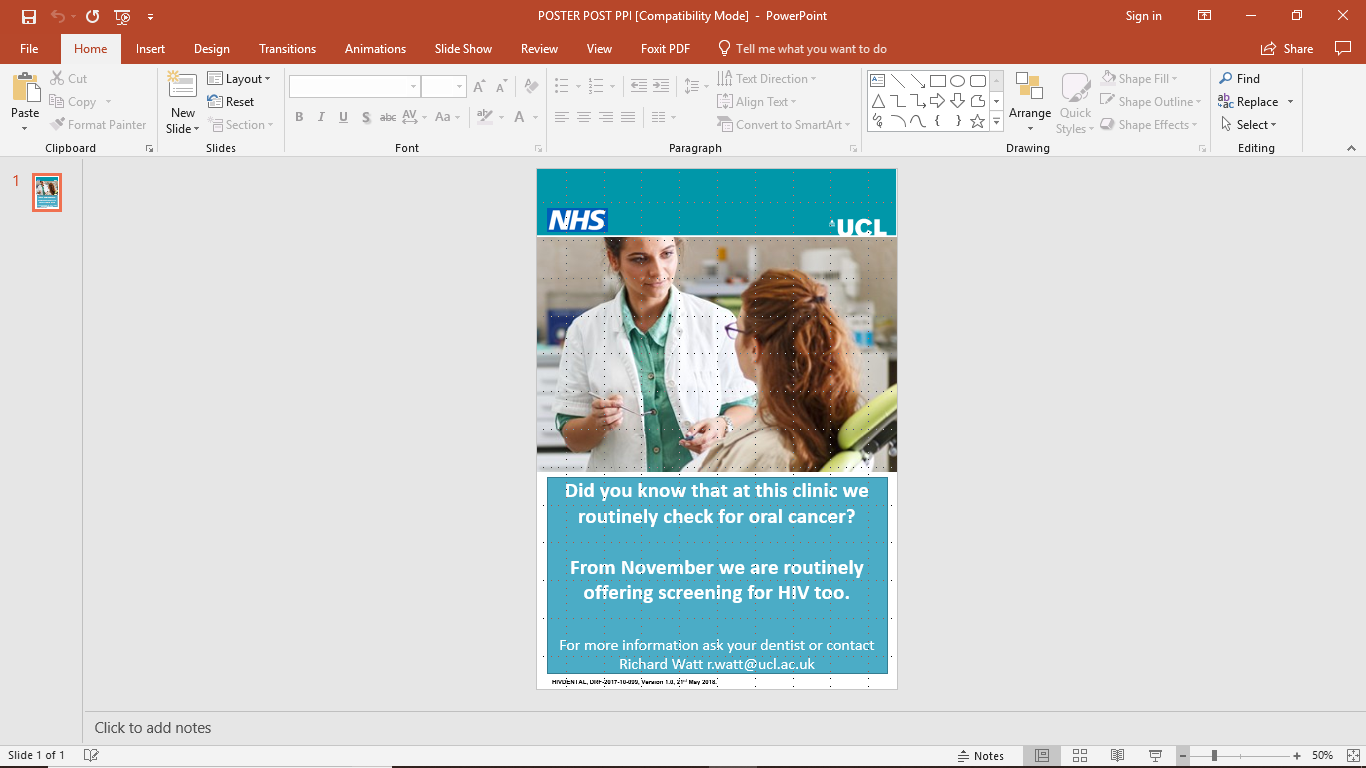

Supplement: Supplementary file 1 [file Datasheet1.docx]
